# Physiological, Biochemical, and Molecular Analyses Reveal Dark Heartwood Formation Mechanism in *Acacia melanoxylon*

**DOI:** 10.3390/ijms25094974

**Published:** 2024-05-02

**Authors:** Ruping Zhang, Xiaogang Bai, Zhaoli Chen, Mengjiao Chen, Xiangyang Li, Bingshan Zeng, Bing Hu

**Affiliations:** Key Laboratory of State Forestry and Grassland Administration on Tropical Forestry, Research Institute of Tropical Forestry, Chinese Academy of Forestry, Guangzhou 510520, China; zrpzrpzrp1@outlook.com (R.Z.); bxg469808@163.com (X.B.); zlchen0525@163.com (Z.C.); cmjmengjiao1997@163.com (M.C.); lixiangyang2019@caf.ac.cn (X.L.)

**Keywords:** *Acacia melanoxylon*, clones, flavonoids, heartwood formation, metabolomic, transcriptomic, phenolics

## Abstract

*Acacia melanoxylon* is highly valued for its commercial applications, with the heartwood exhibiting a range of colors from dark to light among its various clones. The underlying mechanisms contributing to this color variation, however, have not been fully elucidated. In an effort to understand the factors that influence the development of dark heartwood, a comparative analysis was conducted on the microstructure, substance composition, differential gene expression, and metabolite profiles in the sapwood (SW), transition zone (TZ), and heartwood (HW) of two distinct clones, SR14 and SR25. A microscopic examination revealed that heartwood color variations are associated with an increased substance content within the ray parenchyma cells. A substance analysis indicated that the levels of starches, sugars, and lignin were more abundant in SP compared to HW, while the concentrations of phenols, flavonoids, and terpenoids were found to be higher in HW than in SP. Notably, the dark heartwood of the SR25 clone exhibited greater quantities of phenols and flavonoids compared to the SR14 clone, suggesting that these compounds are pivotal to the color distinction of the heartwood. An integrated analysis of transcriptome and metabolomics data uncovered a significant accumulation of sinapyl alcohol, sinapoyl aldehyde, hesperetin, 2′, 3, 4, 4′, 6′-peptahydroxychalcone 4′-O-glucoside, homoeriodictyol, and (2S)-liquiritigenin in the heartwood of SR25, which correlates with the up-regulated expression of *CCRs* (*evm*.*TU*.*Chr3*.*1751*, *evm*.*TU*.*Chr4*.*654_667*, *evm*.*TU*.*Chr4*.*675*, *evm*.*TU*.*Chr4*.*699*, and *evm*.*TU*.*Chr4*.*704*), *COMTs* (*evm*.*TU*.*Chr13*.*3082*, *evm*.*TU*.*Chr13*.*3086*, and *evm*.*TU*.*Chr7*.*1411*), *CADs* (*evm*.*TU*.*Chr10*.*2175*, *evm*.*TU*.*Chr1*.*3453*, and *evm*.*TU*.*Chr8*.*1600*), and *HCTs* (*evm*.*TU*.*Chr4*.*1122*, *evm*.*TU*.*Chr4*.*1123*, *evm*.*TU*.*Chr8*.*1758*, and *evm*.*TU*.*Chr9*.*2960*) in the TZ of *A*. *melanoxylon*. Furthermore, a marked differential expression of transcription factors (TFs), including *MYBs*, *AP2/ERFs*, *bHLHs*, *bZIPs*, *C2H2s*, and *WRKYs*, were observed to be closely linked to the phenols and flavonoids metabolites, highlighting the potential role of multiple TFs in regulating the biosynthesis of these metabolites and, consequently, influencing the color variation in the heartwood. This study facilitates molecular breeding for the accumulation of metabolites influencing the heartwood color in *A*. *melanoxylon*, and offers new insights into the molecular mechanisms underlying heartwood formation in woody plants.

## 1. Introduction

Blackwood (*Acacia melanoxylon* R. Br.), which belongs to the *Acacia* genus of the Fabaceae family, is extensively utilized in various fields, such as the decoration, erosion control, habitat, and landscaping fields, because of its multifaceted applications [1,2,3]. In 1964, IAWA (International Association of Wood Anatomists) defined heartwood (HW) as “the inner layer of wood, in the growing trees, there are no living cells, and the reserved substances in it have been removed or converted into heartwood substances”. Additionally, sapwood (SW) was defined as “the part of wood in a living tree that contains living cells and reserve substances”. The transition zone (TZ) was defined as “the inner layer of sapwood, which is the transition between sapwood and heartwood in color and general characteristics”. The heartwood of blackwood is considered as a high-grade timber for making furniture because of its attractive brown/black color, remarkable texture, and durability [2]. Recent studies have also highlighted its increased acoustic emission (AE) signals, positioning *A*. *melanoxylon* as a premium material for musical instruments because of its superior acoustic properties [4]. Generally, the color of heartwood plays a pivotal role in determining its economic value, with darker heartwood being more valuable [2,5]. In Heyuan City, China, various clones of *A*. *melanoxylon* have been selected, each with unique attributes. SR14 is noted for its higher heartwood ratio [6], whereas SR25 is distinguished by its darker heartwood color. Therefore, exploring the metabolites associated with the color differences and underlying genetic regulatory mechanisms in *A*. *melanoxylon* could significantly improve its quality at the breeding stage.

The transformation of sapwood into heartwood in woody plants is typically accompanied by color changes. Being naturally darker, the heartwood of some precious woody trees is more valuable than the sapwood [7,8,9]. The transformation of sapwood into heartwood involves a series of physiological and biochemical changes, including the death of parenchyma cells, the depletion of starch, a substantial increase in secondary metabolites, a significant reduction in water content, and an increase in gas volume [10]. Several studies have linked the accumulation of secondary metabolites (or crucial compounds) in heartwood to its color development [11,12,13]. For example, polyphenolic compounds were found to be vital extractives for heartwood formation in *Cunninghamia lanceolate* [14], while terpenoids were accumulated in the heartwood of *Santalum album* [11,15]. In *Phoebe zhennan*, 20 phenylpropanoids, including cinnamic acids and their derivatives, coumarin acid derivatives, and flavonoids, have been identified as being integral to the golden thread [16]. Additionally, a few reports have discussed variations in heartwood extracts across various tree species. The glycosylation products of luteolin are believed to play a crucial role in regulating the heartwood color of red-heart Chinese fir compared to white-heart Chinese fir [13]. However, there is limited documentation of the variations in heartwood color among different clones lacking white heartwood.

The process of dark heartwood formation is accompanied by a complex gene regulatory network [17,18]. The transition zone between sapwood and heartwood is generally regarded as the critical region for heartwood formation research because of the absence of living cells in heartwood [19]. Several metabolites regulated by curial genes are closely related to the formation of dark heartwood [20]. Previous studies have found that the gene expression of chalcone synthase (*CHS*), flavanone 3-hydroxylase (*F3H*), dihydroflavonol 4-reductase (*DFR*), and phenylalanine ammonia-lyase (*PAL*) in the transition zone may be strongly correlated with the contents of flavonoids and phenolics [18,21]. Moreover, chalcone synthase (*CHS*) and chalcone isomerase (*CHI*) in the transition zone were also found to be related to the difference in heartwood color between the two clones [13]. Additionally, lignin biosynthesis genes are activated during heartwood formation in *Pinus sylvestris* and *Taiwania cryptomerioides* [12,19]. In addition to these differentially expressed genes (DEGs), several transcription factors (TFs) have also been demonstrated to have significant associations with various heartwood extractives [15,19,20]. Several TFs, such as *SBP*, *WRKY*, *C2H2*, and *GARP-G2-like*, have shown strong positive correlations with terpenoid metabolites [13,20]. The up-regulation of TFs (e.g., *MYB* and *bHLH*) was found to be related to increased phenylpropanoids and flavonoids in the golden thread [16].

Given that SR14 and SR25 are clones with high heartwood ratios and volume contents [6], the specific extractives responsible for the color variation in heartwood among different clones of *A*. *melanoxylon* remain largely unexplored. Furthermore, the molecular mechanisms underlying the color variation in dark heartwood among different clones are not yet well understood. In this study, we employed microscopic observation and biochemical determination to characterize metabolite variations among the SW, TZ, and HW of *A*. *melanoxylon*. By integrating indicator metabolites with DEGs, we utilized transcriptomic and metabonomic approaches to elucidate the regulatory relationship between genes and metabolites in SR14 and SR25. Our research provides new insights into the molecular mechanisms that drive color variation in *A*. *melanoxylon* heartwood and suggests the potential to improve heartwood quality through the molecular breeding of this species.

## 2. Results

### 2.1. Different Colors and Heartwood Compounds Were Observed in SR14 and SR25 through Microscopic Examination

**Figure 1:**
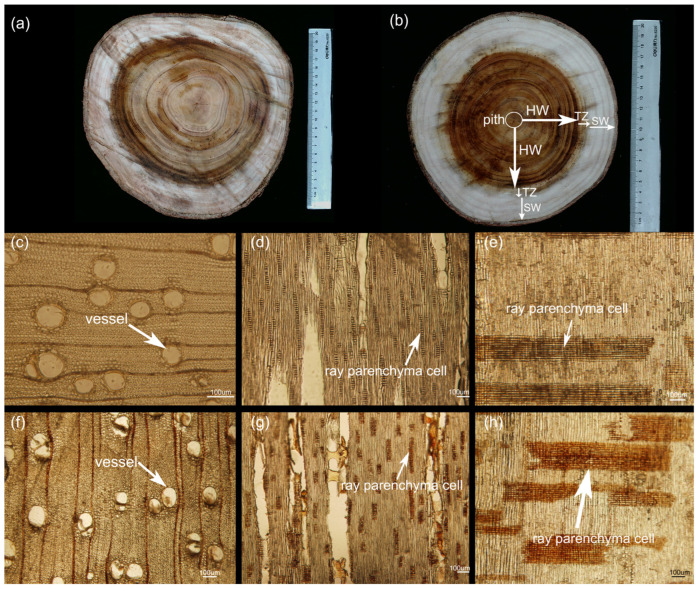
The microstructure of heartwood was observed from the cross-section, radial section, and tangent section and compared between SR14 and SR25 clones. Note, (**a**,**b**) belongs to the polished cores at DBH of SR14 and SR25, respectively, (**c**–**e**) and (**f**–**h**) belong to the heartwood of SR14 and SR25 observed under the microscope from cross-section, radial section, and tangent section, respectively. Scale bars = 10 µm.

To determine the differences in heartwood color, we selected samples from three locations at the breast height of two typical clones (SR14 and SR25) for a comparison. The results indicate that SR14 and SR25 exhibited golden brown and walnut hues in their heartwood, respectively, with the sapwood generally displaying a pale cream color (Figure 1a,b). The heartwood color of SR25 was significantly darker than that of SR14, with no observable difference in the sapwood color of the two clones (Figure 1a,b).

To further investigate the microscopic basis for this color distinction, we prepared thin cross-sections of heartwood tissue for a detailed examination. From the comparative results of the transverse section and the chordal section, it was found that the content of compounds in the vessels from the heartwood of SR25 was significantly higher than that of SR14 (Figure 1c,d,f,g). In addition, the color of the ray parenchyma cells from the stem section and chord section was darker in SR25 than in SR14 (Figure 1d,e,g,h). Interestingly, the content of tyloses in the ray parenchyma cells was higher in the heartwood of SR25 than in that of SR14 (Figure 1). Hence, we hypothesized that the variation in heartwood color is intricately linked to the composition of compounds within the ray parenchyma cells.

### 2.2. Determination of Crucial Compounds Showed Variations in SW, TZ, and HW of SR14 and SR25

**Figure 2:**
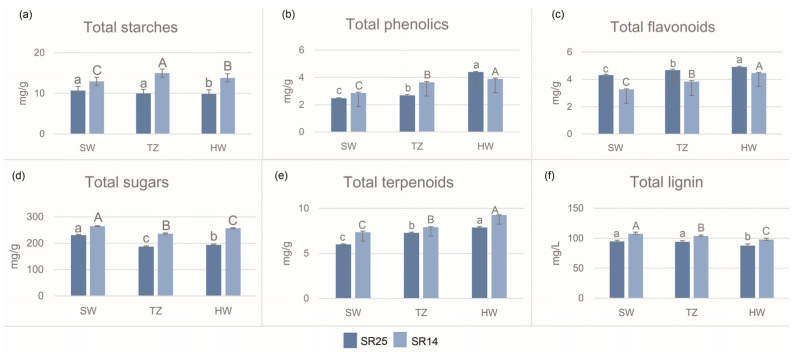
Determination of six compounds (starches, phenolics, flavonoids, sugars, and terpenoids) in SW, TZ, and HW of SR14 and SR25 clones. Note: (**a**–**f**) are the comparison of the contents of total starches, total phenolics, total flavonoids, total sugars, total terpenoids and total lignin among different clones,respectively. Data are presented as the mean ± SE. Different capital letters indicate that the treatment effect is significantly different at the *p* < 0.05 level. Lowercase letters and uppercase letters belong to clones SR25 and SR14, respectively.

Color changes typically coincide with heartwood formation, and prior research has established a correlation between starches, sugars, and terpenoids and this process. Additionally, genes associated with the biosynthesis of lignin have been established as influential factors in the heartwood formation process. Our measurement results showed that the contents of phenols, flavonoids, and terpenoids were higher in the HW than in the SW and TZ, regardless of whether they were in SR25 or SR14 (Figure 2b,c,e). However, the levels of starches, sugars, and lignin were consistently higher in the SW than in the HW and TZ, across both SR25 and SR14 (Figure 2a,d,f).

Consistent with our expectations, the concentrations of phenols and flavonoids, key metabolites that impact heartwood development, were observed to be greater in the heartwood of SR25 than in that of SR14 (Figure 2b,c). It is worth noting that the contents of terpenoids and lignin in the heartwood of SR14 were higher than in SR25, regardless of whether it was in the SW, the TZ, or the HW (Figure 2b,c). The results for the crucial compounds correspond to the results of the tissue microscopic observation, suggesting a correlation between phenols and flavonoids and the variations in heartwood color among *A*. *melanoxylon* clones.

### 2.3. Screening of Differential Metabolites between SR14 and SR25 Using UPLC/HRMS

**Figure 3:**
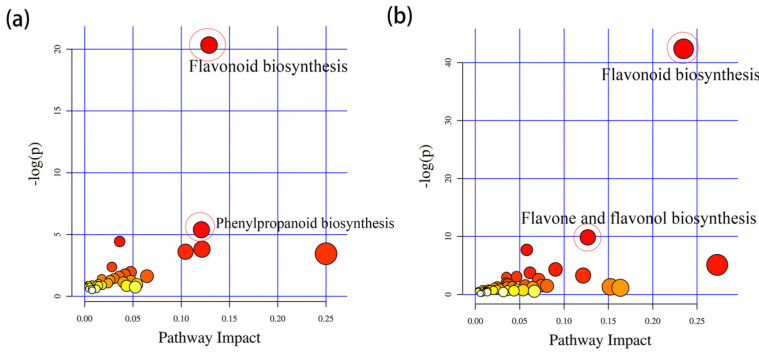
The KEGG analysis of differentially expressed metabolites in SR25HW vs. SR14HW and SR25SW vs. SR14SW groups for *A*. *melanoxylon*. Note, (**a**) belongs to the KEGG enrichment of SR25HW vs. SR14HW group; figure (**b**) belongs to the KEGG enrichment of SR25SW vs. SR14SW group. Note, the color of the circle represents the q value, which is the *p* value after multiple hypothesis test correction. The darker the color, the more reliable the enrichment significance of differentially expressed genes in this pathway. The size of the circle indicates the number of genes enriched in the pathway, and the larger the circle, the more genes there are.

A multivariate statistical analysis showed that R2 and Q2 were higher than 0.5, indicating that the repeatability of the samples was good (Table S1). The metabolites in the sapwood and heartwood and those within the clones were divided into two groups in a PCA (Principal Component Analysis) (Figure S1), which indicated that there were differences in metabolite accumulation between the sapwood and heartwood of various clones. Among all of the differentially accumulated metabolites (DAMs), the number of metabolites identified in the positive-ion mode was higher than that identified in the negative-ion mode (Figure S2 and Table S1). Additionally, 2015 up-regulated and 1752 down-regulated metabolites were identified in the positive-ion mode in the sapwood of the two clones, and 1059 up-regulated and 2219 down-regulated metabolites were identified in the positive-ion mode in the transition zone of the two clones (Figure S2 and Table S1). The DEMs (differentially enriched metabolites) were further annotated using the Kyoto Encyclopedia of Genes and Genomes (KEGG) database. The results of the network diagram in heartwood between two clones showed that flavonoid biosynthesis and phenylpropanoid biosynthesis were the main significant enriched pathways (Figure 3a). In addition, the results for the sapwood between two clones showed that flavonoid biosynthesis, flavone, and flavonol biosynthesis were the main significant enriched pathways (Figure 3b).

### 2.4. Primary DAMs Related to the Formation of Dark Heartwood in SR14 and SR25 Based on Metabonomic Analysis

**Figure 4:**
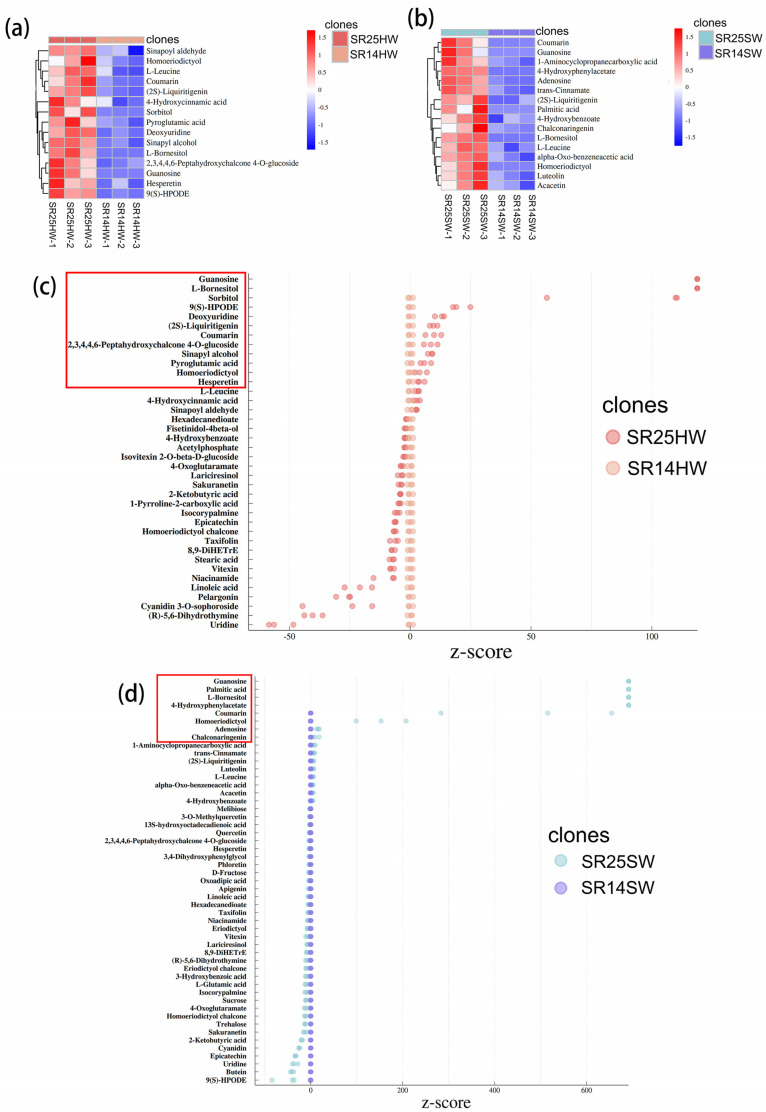
The analysis of cluster thermogram and Z-score (standard score) for differentially expressed metabolites in SR25HW vs. SR14HW and SR25SW vs. SR14SW groups for *A*. *melanoxylon*. Note, (**a**,**c**) belongs to the SR25HW vs. SR14HW group; figure (**b**,**d**) belongs to the SR25SW vs. SR14SW group. The deeper the color scale in the cluster diagrams of (**a**,**b**), the higher the accumulation level of differential metabolites which are significantly up-regulated or down-regulated. The metabolites in the red box are quite different.

To identify metabolites associated with heartwood color among the different clones of *A*. *melanoxylon*, we performed clustering and statistical analyses of metabolites (Figure 4). Based on FC > 1, VIP ≥ 1, and a *p*-value < 0.05, we constructed a clustering heatmap, and then 15 and 16 significant DAMs were identified in the heartwood of SR25 and SR14 and in the sapwood of SR25 and SR14, respectively (Figure 4a,b and Table S2). Notably, most of these metabolites were classified as flavonoids (Table S2). Next, based on the Z-score (standard score) method, twelve and eight metabolites with a higher content in the SR25 clone were screened in the comparison groups of SR25HW vs. SR14HW and SR25SW vs. SR14SW, respectively (Figure 4c,d). Finally, based on the ROC results (receiver operating characteristic curve), ten metabolites with a high content in the heartwood of SR25—sorbitol; 2′, 3, 4, 4′, 6′-peptahydroxychalcone 4′-O-glucoside; hesperetin; 9(S)-HPODE; homoeriodictyol; (2S)-Liquiritigenin; sinapyl alcohol; coumarin; guanosine; and L-bornesitol—were selected as potential metabolite markers to explain the darker color of the heartwood of SR25 (Figure S3). Of these ten metabolites, five metabolites belong to flavonoid biosynthesis pathways (2′, 3, 4, 4′, 6′-peptahydroxychalcone 4′-O-glucoside; hesperetin; homoeriodictyol; (2S)—liquiritigenin; and sinapyl alcohol). Meanwhile, three metabolites (adenosine, homoeriodictyol, and guanosine) affecting the formation of dark-colored sapwood in the SR25 clone were also identified from the ROC curve (Figure S3).

Similarly, based on the clustering and Z-score results, eight DEMs—9(S)-HPODE; 1-Aminocyclopropanecarboxylic acid; uridine; adenosine; 2′, 3, 4, 4′, 6′-peptahydroxychalcone 4′-O-glucoside; 4-Oxoglutaramate; isocorypalmine; acetylphosphate; and cyanidin 3-O-sophoroside—were identified to be up-regulated in the heartwood of the SR14HW vs. SR14SW group (Figure S4a,b). Additionally, ten DEMs—1-Aminocyclopropanecarboxylic acid; trehalose; 9(S)-HPODE; adenosine; 2′, 3, 4, 4′, 6′-peptahydroxychalcone 4′-O-glucoside; uridine; epicatechin; isovitexin 2-O-beta-D-glucoside; isocorypalmine; and sinapoyl aldehyde—were identified to be up-regulated in the heartwood of the SR25HW vs. SR25SW group (Figure S4c,d). Among them, five metabolites—1-Aminocyclopropanecarboxylic acid, 9(S)-HPODE, uridine, adenosine, and isocorypalmine—were typical in two groups (SR25HW vs. SR25SW and SR14HW vs. SR14SW) (Figure S4a–d). Moreover, three metabolites—2′, 3, 4, 4′, 6′-peptahydroxychalcone 4′-O-glucoside; 9(S)-HPODE; and sinapoyl aldehyde—were common in two groups (SR25HW vs. SR25SW and SR25HW vs. SR14HW) (Figure S4c,d and Figure 4a,c). These metabolites may be related to the color difference of the heartwood.

### 2.5. Transcriptomic Signatures and Analysis of DEGs between SW and TZ of SR14 and SR25

**Figure 5:**
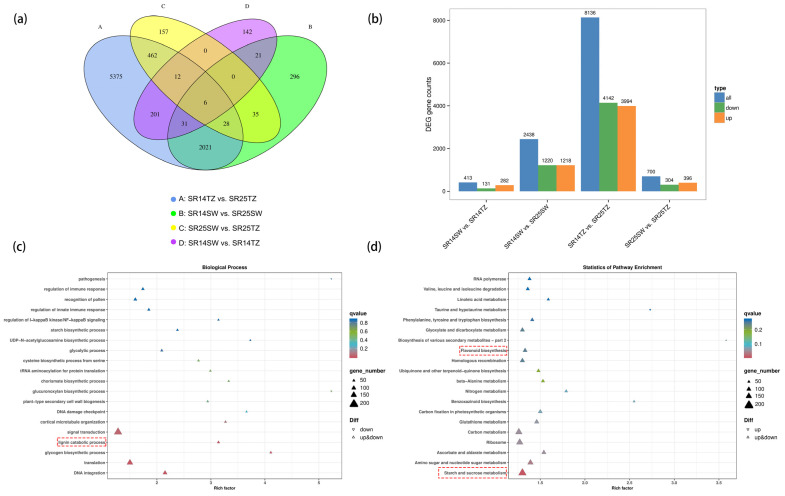
Transcriptomic analysis of differentially expressed genes in four comparison groups (SR14SW vs. SR14TZ, SR14SW vs. SR25SW, SR14TZ vs. SR25TZ, SR25SW vs. SR25TZ) of *A*. *melanoxylon*. (**a**) Venn diagram of DEGs in four comparison groups. (**b**) GO enrichment analysis of D.E.G.s. (**c**,**d**) The GO (the category of molecular function) and KEGG enrichment analysis of DEGs in SR14TZ vs. SR25TZ group, respectively. The pathway in the red box in the picture is our concern.

The DEGs of the SR14TZ vs. SR25TZ group accounted for the majority (8136 DEGs) of the 8797 DEGs from the four comparisons (Figure 5a and Table S3), indicating that significantly different biological events occurred in the transition zones of the two clones. Moreover, the results clearly indicate that the transition zones of the two clones exhibited the highest number of differentially expressed genes, with the most significant disparity being between the up-regulated and down-regulated genes (Figure 5b). A total of 3994 up-regulated genes and 4142 down-regulated genes were found in the SR14TZ vs. SR25TZ group, which may be involved in the formation of the heartwood color of *A*. *melanoxylon* (Table S3). Of these DEGs, 6563 genes were annotated in the SR14TZ vs. SR25TZ group using eight databases (Table S4). The 6563 DEGs were further enriched using Gene Ontology (GO) and KEGG analyses. The results of GO indicated that the “lignin catabolic process (GO: 0046274)” pathway was significantly enriched in the biological process category (Figure 5c). The KEGG analysis revealed significant enrichment in the “starch and sucrose metabolism” pathway, along with enrichment in the “flavonoid biosynthesis” pathway (Figure 5d).

### 2.6. Regulatory Network of Phenylpropanoid and Flavonoid Biosynthesis Pathways Is Related to the Formation of Dark Heartwood

**Figure 6:**
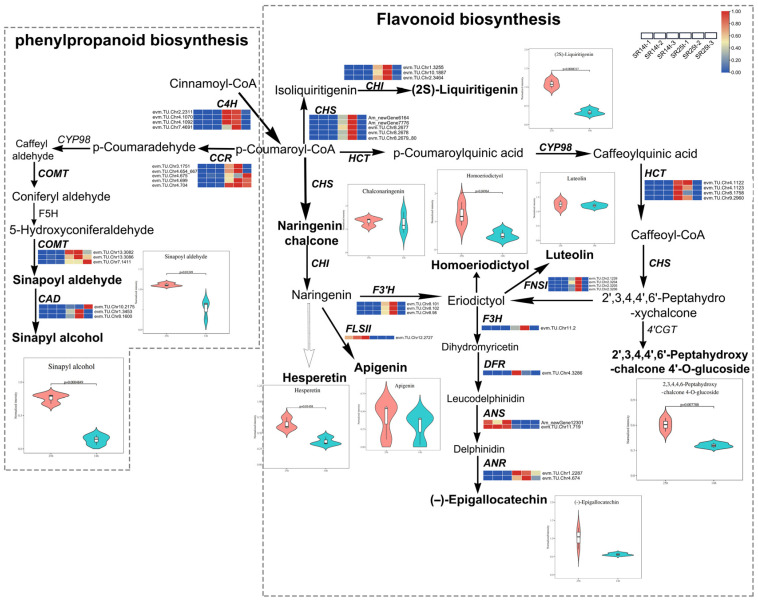
Joint analysis of DEMs and DEGs related to flavonoid and phenylpropanoid biosynthesis pathways. Note, C4H: trans-cinnamate 4-monooxygenase; CCR: cinnamoyl-CoA reductase; 4HA: 4-Hydroxycinnamyl aldehyde; F5H: ferulate-5-hydroxylase; COMT: caffeic acid 3-O-methyltransferase; CAD: cinnamyl-alcohol dehydrogenase; CHS: chalcone synthase; HCT: shikimate O-hydroxycinnamoyltransferase; CYP98A: 5-O-(4-coumaroyl)-D-quinate 3′-monooxygenase; CHI: chalcone isomerase; FLSII**:** flavone synthase II; F3′H: flavanone 3′-hydroxylase; F3H: naringenin 3-dioxygenase; FNSI: flavone synthase I; DFR: Bifunctional dihydroflavonol 4-reductase/flavanone 4-reductase; ANS: Anthocyanin synthase; ANR: anthocyanidin reductase; 4′CGT: chalcone 4′-O-glucosyltransferase. The color scale (0~1) of the heatmap is the normalization of the expression of the same gene.

The results above indicate that the metabolites that exhibit significant differences primarily pertain to flavonoids. The outcomes of the DEGs from the KEGG and GO analyses further confirm the enrichment of the flavonoid biosynthesis pathway and lignin catabolic process in the transition zone of the two clones under investigation. Therefore, we combined the location distribution of differential metabolites and genes, focused on the flavonoid biosynthesis pathway, and drew a joint analysis diagram of genes and metabolites (Figure 6 and Table S5). Clustering heat maps were used to display the differential expression of the genes of the two clones, and violin plots were used to display the differential accumulation of metabolites.

From the overall flavonoid biosynthesis pathway in Figure 6, it can be seen that ten DEMs accumulated more in the heartwood of SR25 than in that of SR14, among which the accumulation of four metabolites [hesperetin; 2′, 3, 4, 4′, 6′-peptahydroxychalcone 4′-O-glucoside; homoeriodictyol; and (2S)-Liquiritigenin] was significantly different. The results of the phenylalanine biosynthesis showed that there were also two significant differential metabolites (sinapyl alcohol and sinapoyl aldehyde). As shown in Table S5, the *CHSs* (*Am_newGene_6164*, *Am_newGene_7776*, *evm*.*TU*.*Chr8*.*2677*, *evm*.*TU*.*Chr8*.*2678* and *evm*.*TU*.*Chr8*.*2679_2680*) and *CHIs* (*evm*.*TU*.*Chr1*.*3255*, *evm*.*TU*.*Chr10*.*1887* and *evm*.*TU*.*Chr2*.*3464*), the upstream genes of the flavonoid biosynthesis pathway, were significantly up-regulated by 9-125 and 5-138 FC (fold change) in the transition zone of SR25, respectively, which may have caused the increase in 2S-Liquiritigenin in the heartwood of SR25. On the one hand, under the up-regulation of the *HCTs* genes, p-coumaroyl caused a remarkable accumulation of the metabolites 2′, 3, 4, 4′, 6′-peptahydroxychalcone 4′-O-glucoside and chalconaringenin in the SR25 heartwood. On the other hand, the *C4Hs* (*evm*.*TU*.*Chr2*.*2311*, *evm*.*TU*.*Chr4*.*1070*, *evm*.*TU*.*Chr4*.*1092*, and *evm*.*TU*.*Chr7*.*4691*), *CCRs* (*evm*.*TU*.*Chr3*.*1751*, *evm*.*TU*.*Chr4*.*654_667*, *evm*.*TU*.*Chr4*.*675*, *evm*.*TU*.*Chr4*.*699*, and *evm*.*TU*.*Chr4*.*704*), *COMTs* (*evm*.*TU*.*Chr13*.*3082*, *evm*.*TU*.*Chr13*.*3086*, and *evm*.*TU*.*Chr7*.*1411*), and *CADs* (*evm*.*TU*.*Chr10*.*2175*, *evm*.*TU*.*Chr1*.*3453*, and *evm*.*TU*.*Chr8*.*1600*), which were significantly up-regulated in the phenylalanine biosynthesis pathway, were up-regulated by 5–54, 73–1745, 3–7, and 2–1043 times, respectively, in the transition zone of SR25, which caused the high accumulation of sinapyl alcohol and sinapoyl aldehyde in the heartwood of SR25. *CHIs* (*evm*.*TU*.*Chr1*.*3255*, *evm*.*TU*.*Chr10*.*1887*, and, *evm*.*TU*.*Chr2*.*3464*) and *F3′Hs* (*evm*.*TU*.*Chr8*.*101*, *evm*.*TU*.*Chr8*.*102*, and *evm*.*TU*.*Chr8*.*98*), as downstream genes of the flavonoid biosynthesis pathway, were up-regulated by 5–138 and 24–2839 times, respectively, in the transition zone of SR25 compared to in that of SR14, which also led to a significantly high accumulation of hesperetin and homoeriodictyol in the heartwood of SR25. Further, the up-regulation of four gene families, *FLS* (*evm*.*TU*.*Chr2*.*1239*, *evm*.*TU*.*Chr2*.*3204*, *evm*.*TU*.*Chr2*.*3205*, and *evm*.*TU*.*Chr2*.*3206*), *DFR* (*evm*.*TU*.*Chr4*.*3286*), *F3H* (*evm*.*TU*.*Chr11*.*2*), and *ANR* (*evm*.*TU*.*Chr1*.*2287* and *evm*.*TU*.*Chr4*.*674*), along with the down-regulation of two gene families, *FLSII* (*evm*.*TU*.*Chr12*.*2727*) and *ANS* (*Am_newGene12301* and *evm*.*TU*.*Chr11*.*719*), led to a higher accumulation of apigenin and (−)-Epigallocatechin in the SR25 heartwood than in the SR14 heartwood.

### 2.7. TFs Related to the Formation of Dark Heartwood

**Figure 7:**
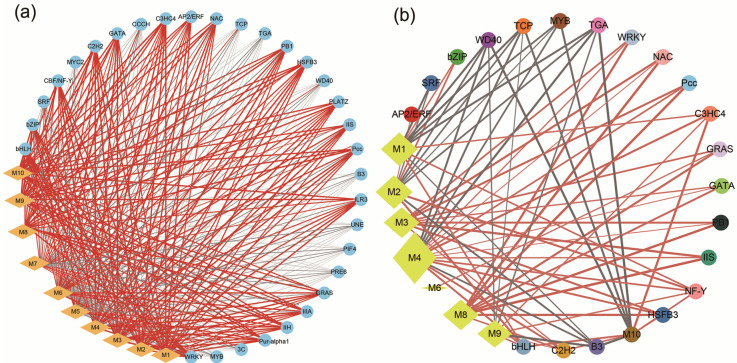
Correlation network diagram between TFs and metabolites in different clones of *A*. *melanoxylon*. Note: (**a**) shows the correlation between metabolites and all differentially expressed transcription factor families (|PCC| ≥ 0.8), and (**b**) shows differentially expressed genes and metabolites with significant correlation (*p* < 0.05 and |PCC| ≥ 0.8). The yellow diamond is a metabolite, and the circles of different colors are different TF. The yellow diamond size represents the number of TFs connected with it. The red line is positively correlated, the gray line is negatively correlated, and the line thickness represents the correlation. M1, 2′, 3, 4, 4′, 6′-peptahydroxychalcone 4′-O-glucoside; M2, Hesperetin; M3, Homoeriodictyol; M4, (2S)-Liquiritigenin; M5, Apigenin; M6, Luteolin; M7, Chalconaringenin; M8, (−)-Epigallocatechin; M9, Sinapoyl aldehyde; M10, Sinapyl alcohol.

A total of 275 differentially expressed transcription factors (DETFs) were identified in the SR14TZ vs. SR25TZ group. These TFs were clustered into thirty-two families, with the top seven families by number of transcripts being *MYB* (62), *AP2/ERF* (46), *WRKY* (37), *bHLH* (24), *SRF* (15), *bZIP* (13), and *C2H2* (12) (Figure 7a and Table S6). The TFs of the *MYBs* (*evm*.*TU*.*Chr4*.*726*, *evm*.*TU*.*Chr7*.*3148*, *evm*.*TU*.*Chr5*.*601*, *evm*.*TU*.*Chr8*.*1926*), five *AP2/ERFs* (*evm*.*TU*.*Chr5*.*356*, *evm*.*TU*.*Chr10*.*156*, *evm*.*TU*.*Chr3*.*843*, *evm*.*TU*.*Chr8*.*1201*, *evm*.*TU*.*Chr6*.*87*), and one *bHLH* (*evm*.*TU*.*Chr4*.*463*) were up-regulated by more than 500 times in the transition zone of SR25 compared to in SR14 (Table S6). And, three *SRFs* (*evm*.*TU*.*Chr10*.*366*, *evm*.*TU*.*Chr1*.*2627*, *evm*.*TU*.*Chr1*.*1247*), three *TGAs* (*evm*.*TU*.*Chr6*.*2122*, *evm*.*TU*.*Chr10*.*4700*, *evm*.*TU*.*Chr13*.*168*), and one *WD40s* (*Am_newGene7612*) were down-regulated in the TZ of SR25 compared with in that of SR14 (Table S6). Moreover, four *MYBs* and one *bHLH* were up-regulated by more than 500 times in the transition zone of SR25 compared to that of SR14 (Table S6). Additionally, three *SRFs*, three *TGAs*, and one *WD40s* were down-regulated by more than 500 times in the transition zone of SR25 compared to that of SR14 (Table S6).

In addition, all of the DETFs and ten metabolites [2′, 3, 4, 4′, 6′-peptahydroxychalcone 4′-O-glucoside, hesperetin, homoeriodictyol, (2S)-Liquiritigenin, apigenin, luteolin, chalconaringenin, (−)-Epigallocatechin, sinapyl alcohol, and sinapoyl aldehyde] from the regulatory network of DEMs were identified in the correlation network analysis (Figure 7 and Tables S6 and S7). Based on | PCC | ≥ 0.8, we found that most of the transcripts encoding *AP2/ERF*, *WRKY*, *bHLH*, *bZIP*, *CBF/NF-Y*, *C2H2*, *GATA*, *C3HC4*, *NAC*, *PB1*, *HSFB3*, *PLATZ*, *IIS*, *Pcc*, *ILR3*, *GRAS*, *IIIA*, *IIH*, and *Pur-alpha1* showed a positive correlation with the ten DEMs. In contrast, most of the transcripts encoding *MYB*, *SRF*, *3C*, *PRE6*, *PIF4*, *UNE*, *B3*, *WD40*, *TGA*, *TCP*, and *CCCH* were negatively correlated with the ten DEMs (Figure 7a). Based on |PCC| ≥ 0.8 and *p* < 0.05, we found that nine metabolites were significantly related to differentially expressed transcription factors, except for apigenin (Figure 7b). Furthermore, a significant negative correlation was observed between three metabolites (2′, 3, 4, 4′, 6′-peptahydroxychalcone 4′-O-glucoside, hesperetin, and sinapyl alcohol) and the majority of TFs, while the remaining six metabolites exhibited a significant positive correlation (Figure 7b). Similarly, most of the transcripts encoding C2H2, bHLH, AP2/ERF, bZIP, WRKY, NAC, Pcc, C*3HC4*, *GRAS*, *GATA*, *PB1*, *IIS*, *CBF/NF-Y*, and *HSFB3* showed a significantly positive correlation with the ten DEMs. Most transcripts encoding *SRF*, *WD40*, *TCP*, *MYB*, *TGA*, and *B3* showed a significantly negative correlation with the ten DEMs (Figure 7b).

### 2.8. RT-qPCR Analysis

The expression levels of the DEGs in the transition zone of two *A*. *melanoxylon* clones (SR14 and SR25) were detected and analyzed using qRT-PCR (Tables S7 and S8), and the DEG results of the RNA sequence and the RT-PCR showed a strong consistency (Tables S7 and S8). For instance, the expression levels of *WRKY71* and *WRKY47* were 13- and 10-fold higher in SR25 than in SR14, respectively. In the phenylpropenoid biosynthesis pathway, the *CAD* and *PAL* transcription levels were 14 and 11 times higher in SR25, respectively (Figure S5 and Table S9). In addition, *AMY* and *SuSy* from the starch and sucrose metabolism pathways were five and three times higher in SR25 than in SR14, respectively. Moreover, the transcription levels of *HCT* and *F3′H* from the flavonoid biosynthesis pathway were three and seventeen times higher in the transition zone of SR25 than in that of SR14, respectively (Table S10).

## 3. Discussion

### 3.1. Differences in Heartwood Color and Substance Content among Different Clones

Previous research found that the heartwood color of *A*. *melanoxylon* varied from pale cream to straw, golden brown, red-brown, and walnut, while the sapwood color was generally pale cream [22]. In this study, the heartwood color of SR25 proved to be significantly darker than that of SR14, with both SR14 and SR25 exhibiting a heartwood color darker than their sapwood. Similarly, the color difference in blackwood′s heartwood is also apparent in different clones in Jiuzhou Town [6]. Thus, the variation in the color of *A*. *melanoxylon* heartwood is related to both between- and within-tree variations [22,23,24]. These results suggest that, in the future, attention should be paid to the color difference in the selection of excellent clones for *A*. *melanoxylon*, in addition to the heartwood metabolite content [3,25].

Starch, sugar, phenols, and flavonoids are commonly employed in investigations of the formation mechanism of heartwood [13,14,15,19]. Given its high commercial value, does the formation mechanism of dark heartwood also involve these substances? In recent years, a few reports have stated that the contents of starch and phenols are related to the color difference of heartwood [13]. In line with Celedon′s three models, it was observed that flavonoids and phenolics exhibited a higher accumulation in the transition zone and heartwood of A. melanoxylon than in the sapwood [11]. The changes in the starch and sucrose contents in the sapwood, transition zone, and heartwood of clone SR14 are consistent with those of the more easily induced agarwood *Aquilaria sinensis* [26]. The total sugar content of clone SR25 is slightly different, which may be related to the clone category. Our dark-heartwood clone SR25 exhibited a higher flavonoid content than the light-heartwood clone SR14, aligning with previous research on the heartwood color of the red-heart Chinese fir (*Cunninghamia lanceolata*) [13]. Few studies have compared the terpene and lignin contents in clones or families with different heartwood colors. In this study, it is noteworthy that the heartwood of SR14 contained higher levels of terpenes and lignin than that of SR25. In future research, the inclusion of clone categories will allow for a more in-depth investigation into the potential relationship between these two substances and heartwood color.

### 3.2. Essential Secondary Metabolites Affecting the Formation of Dark Heartwood

Secondary metabolites play a vital role in the formation of heartwood color for most woody plants with dark heartwood [9,11,13]. From the observation results for three sections of heartwood, it was found that the dark metabolites of ray parenchyma cells were larger in SR25 than in SR14. As anticipated, the transition zone of plants exhibiting dark heartwood demonstrated a lower abundance of metabolites than the heartwood itself [20,26]. Phenolics and flavonoids, which are common metabolites found in heartwood [7,11], exhibited higher levels in SR25 heartwood than in SR14 heartwood, as indicated by the content analysis results. It is worth mentioning that there was no significant difference in the terpene and lignin contents between the heartwood of the two clones. We speculated that these two compounds may have less effect on the color difference in the heartwood. However, in the metabonomic analysis, half of all of the DEMs that may affect the color difference were from the flavonoid biosynthesis pathway. This is consistent with Yang′s result, which indicated that flavonoid biosynthesis is the pathway that influences wood color [14]. Additionally, 2′, 3, 4, 4′, 6′-peptahydroxychalcone 4′-O-glucoside, 9(S)-HPODE, and sinapoyl aldehyde, three similar DEMs in both the SR25HW vs. SR25SW and SR25HW vs. SR14HW groups, were not only the differential metabolites between the heartwood and sapwood but also the differential metabolites between clones, which seems to be noteworthy for the development of dark heartwood in the future. Our findings indicate that the color discrepancy in the heartwood of *A*. *melanoxylon* is associated with molecular-level variations both within and between trees.

### 3.3. Regulatory Network of Genes and Metabolites for the Formation of Dark Heartwood

The transcripts of flavonoid and phenylpropanoid biosynthesis genes have been proven to be closely related to the content of metabolites that influence color formation [15,16,26,27]. Understanding the regulatory mechanism of these metabolites and genes may help increase the content of the desired compounds at the molecular breeding level in the future [20]. Lignin, abundant in the heartwood of trees, plays a crucial role in enhancing the wood’s hardness, resistance to corrosion, and color development [28,29,30,31], being an intricate polymer consisting of aromatic subunits derived from phenylalanine [32]. Limited research has compared the DEGs related to heartwood color solely within the transition zones associated with phenylpropanoid synthesis. Still, Cao et al. [13] and Lim et al. [19] reported that *CADs* and *COMTs* were more up-regulated in the transition zone than in sapwood. In our results of phenylpropanoid biosynthesis, except for the up-regulation of *C4Hs* and *COMTs*, *CCR* and *CAD* unigenes were up-regulated a thousand times more in the transition zone of SR25 than in that of SR14. Hence, based on phenylpropanoid synthesis, we hypothesized that CCRs and CADs are pivotal genes associated with the variation in heartwood color between the SR25 and SR14 clones. The *CAD* family has been proven to catalyze the synthesis of sinapyl alcohol from sinapoyl aldehyde in several plants [33,34]. Previous research on transgenic poplar and alfalfa has demonstrated that the down-regulation of CCR results in a reduced lignin content, subsequently increasing the availability of fermentable sugars [35,36,37]. A metabolomics analysis revealed markedly elevated levels of sinapyl alcohol and sinapoyl aldehyde in the heartwood of SR25 compared with in that of SR14. Consequently, we hypothesized that the up-regulation of CCRs and CADs in the transition zone may be associated with the heightened abundance of these two metabolites.

However, the results of research on red-heart Chinese fir suggest that luteolin’s glycosylation products might be the essential compounds in regulating the color difference between heartwood and sapwood [13]. Previous research results for *Dalbergia oliveri* showed that three metabolites (isoliquiritigenin, formononetin, and biochanin A) could be the main pigment components affecting the color difference between heartwood and sapwood [38]. Yang et al. selected 21 DEMs (flavonoids, coumarin derivatives, and stilbenes) from a comparison group of HW and SW as critical metabolites related to the darker heartwood of *Cunninghamia lanceolata* [20]. The identified significant DEMs—including hesperetin; 2′, 3, 4, 4′, 6′-pentahydroxychalcone 4′-O-glucoside; homoeriodictyol; and (2S)-liquiritigenin—associated with darker heartwood in this study, exhibit inconsistencies with the findings of previous research. These disparities may stem from variations in tree species and the composition of the comparison groups (SR25HW vs. SR14HW, SR25HW vs. SR25SW, and SR14HW vs. SR14SW). The difference in color-related metabolites could also be related to the differential expression of genes. In a previous study, the results of the wood color of *Taxus chinensis* showed that anthocyanin accumulation was correlated with the expression levels of *DFR*, *ANS*, *F3′H*, and flavonoid 3′,5′-hydroxylase [39]. In a study of sugarcane roots, p-coumaroyl-CoA transferred into homoeriodictyol chalcone due to the up-regulation of the expression of *HCTs*, *CHSs*, and phlorizin synthase [40]. In this study, it was found that, in the regulatory network of flavonoid biosynthesis, in addition to the up-regulation of other genes (*CHS*, *CHI*, *FLS*, *F3H*, *DFR*, and *ANR*), *HCT* and *F3′H* were up-regulated by 7–497 and 24–2839 times, respectively, in the transition zone of SR25 compared with in that of SR14 for *A*. *melanoxylon* (Figure 6). Therefore, the results show strong correlations between the four DEMs and the expression levels of *HCT* and *F3′H*, which were highly significant.

To sum up, the dark heartwood formation of *A*. *melanoxylon* is associated with accumulation levels of specific metabolites (sinapyl alcohol; sinapoyl aldehyde; hesperetin; 2,3,4,6-tetrahydroxychalcone 4′-O-glucoside; homoeriodictyol; and (2S)-Liquiritigenin) in the phenylpropanoid and flavonoid biosynthesis pathways, and they are regulated by the up-regulation of key genes (CCR, CAD, HCT, and F3′H).

### 3.4. TFs Regulate Metabolite Biosynthesis Related to the Formation of Dark Heartwood

Similar to Scots Pine (*Pinus sylvestris*), the transcription factors MYB and NAC in the transition region of the *A*. *melanoxylon* SR25 clone were also significantly differentially expressed [19]. The transcription factors encoding bHLH, WRKY, and AP2/ERF were significantly differentially expressed in the transition zone of the *A*. *melanoxylon* SR25 clone, which is consistent with the research results for the red-heart Chinese Fir [13]. The relationship between transcription factors and secondary metabolites has also been discussed in previous studies. *MYB*, *AP2-ERF*, *bZIP*, *NAC*, *C2C2*, *C2H2*, and *GRAS* were shown to be associated with the production of flavonols/anthocyan in *Narcissus tazetta* [41]. Five TFs, *MYB*, *AP2/ERF*, *bZIP*, and *TCP*, showed significant differential expression in phenylpropanoid synthesis, and they may be related to flavone formation in Purple Tea [27]. SaCYP736A167, as a multi-substrate P450, stereo-selectively produces (Z)-α-santalol, (Z)-β-santalol, (Z)-epi-β-santalol, and (Z)-α-exo-bergamotol, matching authentic sandalwood oil [15]. Are the transcription factors in the transition zone related to the high accumulation of secondary metabolites in heartwood? In this study, the expression of *MYB*, *AP2/ERF*, *bHLH*, *bZIP*, and *C2H2* was up-regulated in the transition zone of SR25, and four TFs (*AP2/ERF*, *bHLH*, *bZIP*, and *C2H2*) showed a significantly positive correlation with phenolics and flavonoids. Research has proven that *WRKY* is also involved in regulating the contents of flavonoids and phenylpropanoids [42]. In this research, *WRKY* had a significantly positive correlation with the biosynthesis of phenolics and flavonoids. Hence, these transcription factors may be associated with the metabolites that control the heartwood color of *A*. *melanoxylon*.

## 4. Materials and Methods

### 4.1. Plant Material

Previous research found that the heartwood colors of ten-year-old clones SR14 and SR25 exhibited significant variation, with a high growth index being noted [6]. Consequently, these two clones were chosen for the current study. In March 2022, samples of the different clones (SR14 and SR25) were collected at DBH (diameter in breast, 1.3 m above the ground), based on the color variation observed in the heartwood, transition zone, and sapwood of individual trees. A schematic diagram illustrating the selection of the SW, HW, and TZ is presented in Figure 1a,b. With the exception of the samples designated for a microscopic analysis, all samples were collected after tree felling, while the remainder were obtained using growth cones without any trees being cut down. A portion of the samples was stored in liquid nitrogen at −80 °C for the analysis of compound contents, RNA extraction, and metabolite extraction. The remaining portion was fixed in FAA solution for a tissue microscopic observation and a staining analysis. All samples (SW, HW, and TZ) were collected from 10-year-old *A*. *melanoxylon* trees in Zhongba Town, Heyuan City, China. There were three biological replicates of both clones, and their cores were drilled at DBH, approximately 1 cm apart. Throughout this manuscript, the labels SR25HW, SR25TZ, and SR25SW, and SR14HW, SR14TZ, and SR14SW represent the heartwood, transition zone, and sapwood of the *A*. *melanoxylon* clones SR25 and SR14, respectively.

### 4.2. Content Detection of Compounds in SW, TZ, and HW of Two Clones

The sample collection method was identical to the one described above. After being crushed and dried to a consistent weight, the stem portions were sieved through a 40-mesh sieve. Starch and sugars were detected using the anthrone colorimetric and miniaturization-DNS methods, respectively [43,44].

The contents of total phenolics, flavonoids, and total terpenes were detected using the micro-method at 760 nm absorbance, micro-method at 470 nm absorbance, and the vanillin–glacial acetic acid colorimetric method at 535 nm absorbance with a spectrophotometer, respectively [43,45,46]. In an alkaline nitrite solution, a red complex is formed by total flavonoids and aluminum ions, exhibiting a distinct absorption peak at 470 nm. Therefore, the total flavonoid content in the samples could be determined by measuring its absorption at 470 nm. Under alkaline conditions, tungstomolybdic acid undergoes reduction by phenols, yielding blue compounds with a distinct absorption peak at 760 nm. Therefore, the total phenol content in the samples could be determined by measuring the absorption value at 760 nm. Additionally, lignin contents were detected using a test kit with the double-antibody sandwich method (Shanghai Jingkang Biological Engineering Co., Ltd., Nanjing, China). There were three biological replicates of both clones and three technical replicates for each. ANOVA was used to carry out a statistical analysis, and *p* < 0.05 was considered to be significantly different.

### 4.3. Microscopic Observation of Heartwood of SR25 and SR14

Three trees of each clone were felled, and 5–7 cm thick discs were sawed at breast height using a sterilized chainsaw. Subsequently, heartwood blocks measuring 1–1.5 cm in length and width and 2.5–3 cm in height were promptly extracted from these discs as raw samples. The raw samples were put into FAA stationary liquid. Next, the samples were rinsed in buffer and then deionized water. Further, the samples were sliced into 15–20 µm sections using a semi-automatic microtome (Leica RM2255, Wetzlar, Germany). Finally, the sections were observed using an optical microscope (OLYMPUS BX51, Tokyo, Japan). Each sample’s cross-section, radial section, and tangential section were photographed at multiple angles. Additionally, five slides of each section (cross-section, radial section, and tangential section) were observed.

### 4.4. Metabolite Extraction and Profiling

Based on a previous study, metabolites were extracted as follows [47]: To begin, 200 mg of the sample, 0.6 mL of methanol with an internal standard, and the necessary glass beads were combined in a tube. The mixture was then shaken for 1 min. Following a 3 min grinding process, ultrasound was applied for 15 min. Then, after centrifugation for 10 min, 200 μL of the supernatant was transferred to a detection bottle. In addition, 20 L of each sample was removed during quality control (QC). A mixed standard curve was prepared as follows: The sample supernatant, obtained through metabolite extraction, was transferred into a 2 mL centrifuge tube and vortexed for 1 min. Subsequently, the sample was diluted with methanol, containing an internal standard, to achieve the desired concentration. Six standard curve points were acquired, and they were arranged to be tested together. Chromatographic separation and mass spectrometry were conducted according to the procedures outlined by Abdelhafez et al. and Monnerat et al. [48,49]. The original data were ultimately acquired by undergoing format transformation [50]. Metabonomic determination was carried out by PANOMIX company (Suzhou PANOMIX biomedical technology Co., Ltd, Suzhou, China).

In order to compare the metabolite variances between SR14 and SR25 through a metabonomic lens, we conducted a UPLC/HRMS analysis on 12 samples of SW and HW from both clones. The QC samples exhibited approximately 70% of the characteristic peaks, with an RSD below 30%, confirming the overall data quality (Table S1). Based on *p*-value ≤ 0.05 and VIP (Variable Importance in Projection) ≥ 1, the differential metabolites of multiple groups were obtained. To analyze the metabolites between the four groups (SR14HW vs. SR14SW, SR25HW vs. SR25SW, SR25SW vs. SR14SW, and SR25HW vs. SR14HW), we conducted multivariate statistical analyses (Principal Component Analysis after adaptive (UV) conversion processing, partial least squares discriminant analysis, and Orthogonal Projections to Latent Structures Discriminant Analysis) to obtain more reliable and intuitive results [51,52,53,54]. In the identification of metabolites, the accuracy of the molecular weight of the metabolites was first confirmed (molecular weight error < 15 ppm), and then the differential metabolites were identified by further matching the annotations in Metlin (https://metlin.scripps.edu, accessed on 22 February 2024) and Mona (https://mona.fiehnlab.ucdavis.edu, accessed on 22 February 2024) according to the fragment information obtained in the MS/MS mode. Additionally, we conducted a hierarchical clustering analysis using the relative values of DEMs, and we visualized the outcomes using the heatmap package in R (version 3.3.2). The Z-score (standard score) was converted based on the relative content of metabolites. KEGG (Kyoto Encyclopedia of Genes and Genomes) was used to determine the enrichment of metabolic pathways [55]. MetPA (www.metaboanalyst.ca, accessed on 22 February 2024) was used to analyze the related metabolic pathways of the different metabolites in each group, and a corresponding network diagram of the metabolic pathways was drawn according to the results.

### 4.5. RNA Extraction and Sequence Analysis

The total RNA was extracted from the SW and TZ using an Aidlab EASY Spin Kit (Aidlab Biotech, Beijing, China). Each clone consisted of three trees, with the SW and TZ also comprising three biological replicates. After the library was constructed, it was initially quantified using a Qubit 3.0 fluorescence quantifier. After the library passed the quality inspection, the PE150 pattern was sequenced using an Illumina NovaSeq6000 sequencing platform by BIOMARKER Company (Biomarker Technologies, Beijing, China). Additionally, the data were mapped to the blackwood genome. Based on the reference genome sequence, the mapped reads were spliced and annotated with the original genome annotation information using the Hisat–StringTie–ballgown method [56]. The KEGG and GO annotation results of the transcriptome sequences were derived from the reference genome.

After the quality control of the sequencing data, 90.01 Gb (Gigabyte) clean data were obtained, and Q30 was no less than 91.75% for each sample (Table S11). The mapping ratio of each sample ranged from 82.49% to 89.90% (Table S12). In addition, 6087 novel genes were discovered, excluding short transcripts or those containing only one exon. In this study, we used StringTie for standardization with the maximum flow algorithm, and FPKM (fragments per kilobase of transcript per million fragments mapped) was used as an index to measure the expression levels of transcripts or genes [57]. Additionally, a PCA (Principal Component Analysis) was used to evaluate the dispersion of samples. DESeq2 software was used to analyze the differential expression of genes based on the condition of fold change ≥ 2 and FDR < 0.01 (statistical significance set at *p* < 0.05) [58]. Transcriptome annotation was based on our genome annotation file, which is yet to be published. The heatmap package in Tbtools v1.045 was used for a differential expression analysis [59]. Based on *p*-value ≤ 0.05 and q-value ≤ 0.05, the topGO (E-value ≤ 1 × 10^−5^) and KEGG (E-value ≤ 1 × 10^−5^) programs in ClusterProfiler (R version 4.1.2) were used for an enrichment analysis. GraphPad Prism 8.0 was used to draw a violin chart to show the difference in metabolite accumulation levels.

In the joint analysis chart of metabolic pathways, besides the cluster diagram (gene expression), the violin chart is also based on ANOVA software for significant analysis of the values. And the experimental data undergo ANOVA analysis, with statistical significance set at *p* < 0.01 and 0.01< *p* < 0.05 in metabolite accumulation level (violin chart), and *p* < 0.05 in gene expression. Adobe Illustrator 2022 is used to splice and integrate pictures.

### 4.6. Correlation Analysis of Metabolites and TFs

The network diagram described the relationship between metabolite intensity and the expression level of TFs was drawn. First, the Pearson correlation coefficient (PCC) was used to calculate the correlation coefficient between genes and metabolites by WGCNA. Second, Excel was used for screening and filtering. Third, based on *p* < 0.05 and |PCC| ≥ 0.8, the TFs—metabolite pairs were selected to draw a co-expression network diagram with Cytoscape 3.9.1 (Cytoscape Consortium, San Diego, CA, USA).

### 4.7. Expression Validation of the Genes with Real-Time Quantitative (RT-PCR)

According to instructions, total RNA was extracted from SW and TZ with an Aidlab R.N.A. extraction Kit (Aidlab Biotech, Beijing, China). Next, the cDNA was synthesized using the Monad cDNA Kit (Monad, Suzhou, China), and a reagent combination for the PCR test was prepared using the SYBR-Green PCR kit (QIAGEN, Hilden, Germany). Then, The ABI7500 (ABI, Los Angeles, CA, USA) was utilized for the qPCR process. Finally, gene expression levels were determined using the 2^−ΔΔCt^ method in triplicate with RPL4 as the internal reference gene [60]. The selection of genes is based on previous studies [61]. Primer3Plus was used for designing primer sequences.

Each value (substance content, gene, and metabolite) in the entire study is derived from three biological replicates. The experimental data underwent ANOVA testing, with statistical significance set at *p* < 0.05.

## 5. Conclusions

The aim of this study was to evaluate the biochemical and molecular underpinnings associated with the formation mechanism of the dark-colored heartwood in *A*. *melanoxylon*, laying the groundwork for the potential genetic enhancement and molecular breeding of heartwood color in subsequent research. The microstructure status, substance content, differential expressed genes, and metabolites were compared among SW, TZ, and HW. The microscopic observation confirmed that the variation in heartwood color correlated with an elevated substance content in ray parenchyma cells. The higher concentrations of phenols and flavonoids in the SR25 clones with dark heartwood compared to SR14 indicate that these substances are crucial in influencing heartwood color discrepancies. Based on metabolite analysis, three pathways and ten significantly differentially expressed metabolites were considered to be related to the color difference of heartwood for *A*. *melanoxylon*. Through transcriptome analysis, three pathways were identified in SR14TZ vs. SR25TZ, comprising 3994 up-regulated genes and 4142 down-regulated genes. By integrating transcriptome and metabolomics data, our analysis suggests a correlation between the increased accumulation of metabolites and the heightened expression of genes in the transition zone. The identified TFs with significant differential expression were associated with phenolic and flavonoid metabolites, suggesting their potential role in regulating the biosynthesis of compounds influencing heartwood color variation. Consequently, there is potential for the modification of these genes and transcription factors in order to increase the production of a variety of flavonoid metabolites that impact the coloration of heartwood, leading to the production of premium dark heartwood.

## Data Availability

Transcriptome sequence data from this article can be found in the NGDC database with the accession number PRJCA020781. The direct link for the NGDC database is https://ngdc.cncb.ac.cn/bioproject/browse/PRJCA020781. In addition, we declare that the genome can be used for assembly and annotation of transcriptome sequencing in this study.

## Supplementary Materials

The following supporting information can be downloaded at: https://www.mdpi.com/article/10.3390/ijms25094974/s1.
