# Supplementary figures and images for: Physiological, Biochemical, and Molecular Analyses Reveal Dark Heartwood Formation Mechanism in Acacia melanoxylon
Source: Int J Mol Sci. 2024 May 2;25(9):4974. doi: 10.3390/ijms25094974 (PMC11084464; doi:10.3390/ijms25094974)

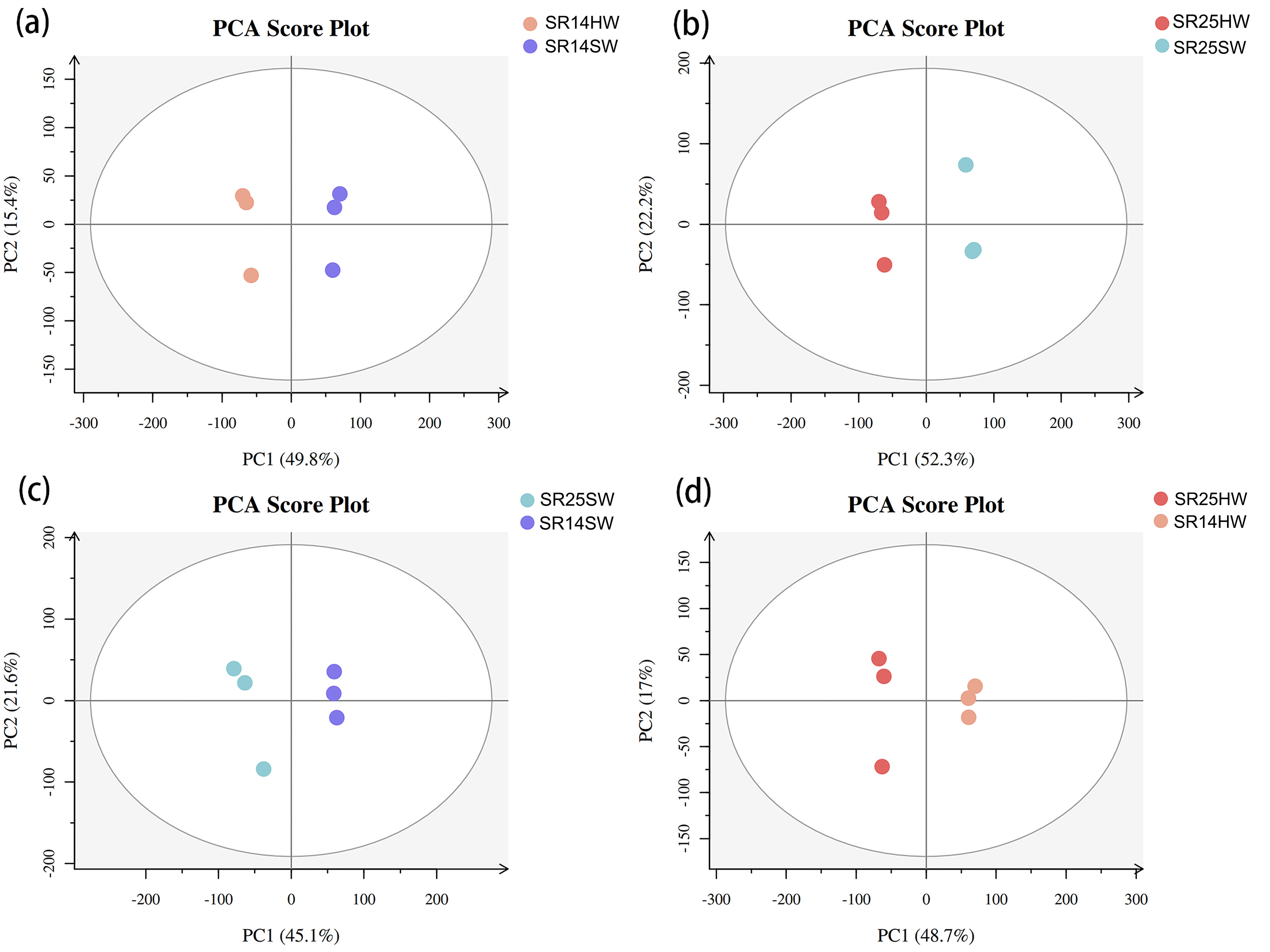

Supplement: Supplementary file 1 [file ijms-25-04974-s001.zip › figureS1.tif]

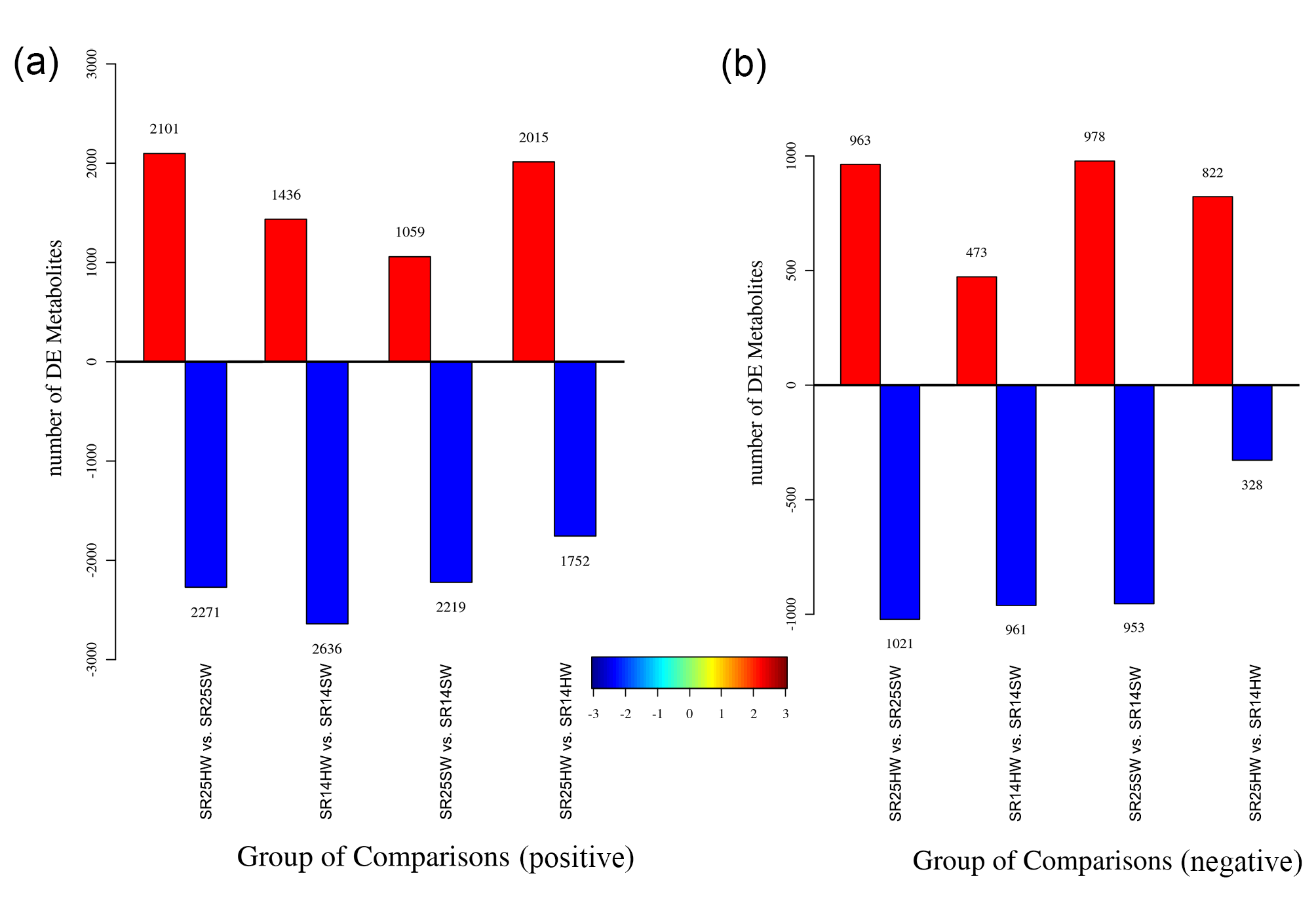

Supplement: Supplementary file 1 [file ijms-25-04974-s001.zip › figureS2.tif]

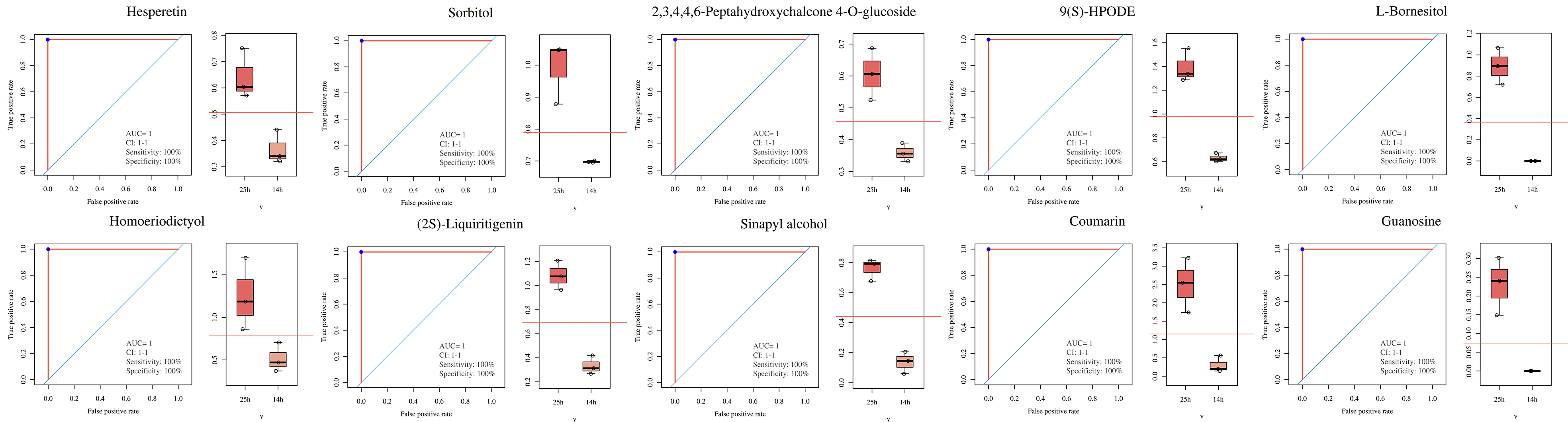

Supplement: Supplementary file 1 [file ijms-25-04974-s001.zip › figureS3.tif]

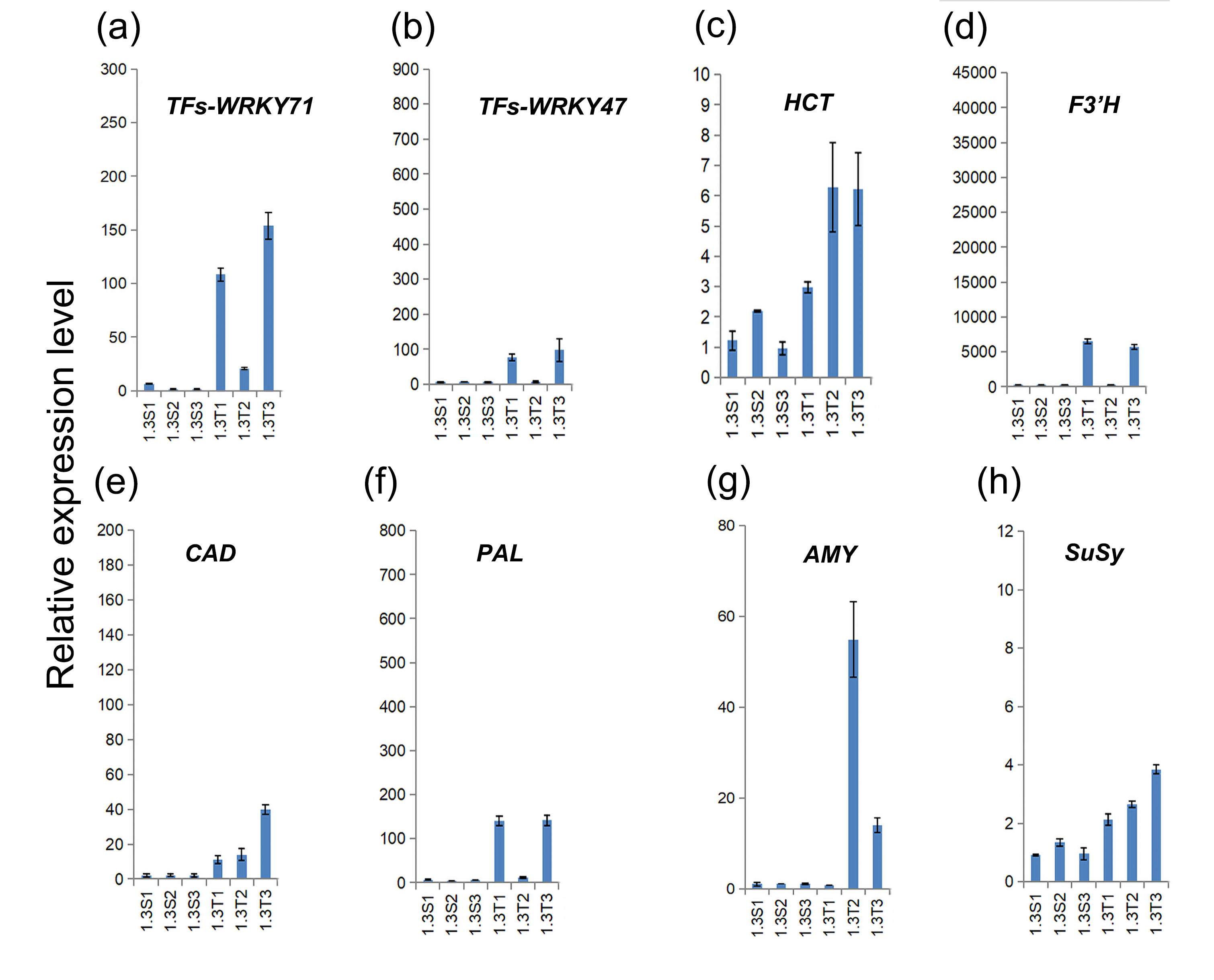

Supplement: Supplementary file 1 [file ijms-25-04974-s001.zip › figureS5.tif]
